# Network meta-analysis of eribulin versus other chemotherapies used as second- or later-line treatment in locally advanced or metastatic breast cancer

**DOI:** 10.1186/s12885-021-08446-8

**Published:** 2021-06-30

**Authors:** Qi Zhao, Rachel Hughes, Binod Neupane, Kristin Mickle, Yun Su, Isabelle Chabot, Marissa Betts, Ananth Kadambi

**Affiliations:** 1 Global Value & Access, Eisai Inc, Woodcliff Lake, NJ USA; 2 Evidence Synthesis, Modeling & Communication, Evidera, San Francisco, CA USA; 3 Evidence Synthesis, Modeling & Communication, Evidera, Montreal, QC Canada; 4 Evidence Synthesis, Modeling & Communication, Evidera, Waltham, MA USA; 5 Global Value & Access, Eisai Inc., Woodcliff Lake, NJ USA; 6 Faculté de pharmacie, University of Montreal, Montreal, QC Canada

**Keywords:** Breast cancer, Metastatic, Locally advanced, Network meta-analysis, Triple negative breast cancer, overall survival

## Abstract

**Background:**

Eribulin mesylate (ERI; Halaven®) is a microtubule inhibitor approved in the United States for metastatic breast cancer patients with at least two prior chemotherapy regimens for metastatic breast cancer, and in the European Union in locally advanced breast cancer or metastatic breast cancer patients who progressed after at least one chemotherapy for advanced disease. This network meta-analysis compared the efficacy and safety of ERI versus other chemotherapies in this setting.

**Methods:**

Systematic searches conducted in MEDLINE, Embase, and the Cochrane Central Register of Clinical Trials identified randomized controlled trials of locally advanced breast cancer/metastatic breast cancer chemotherapies in second- or later-line settings. Efficacy assessment included pre-specified subgroup analysis of breast cancer subtypes. Included studies were assessed for quality using the Centre for Reviews and Dissemination tool. Bayesian network meta-analysis estimated primary outcomes of overall survival and progression-free survival using fixed-effect models. Comparators included: capecitabine (CAP), gemcitabine (GEM), ixabepilone (IXA), utidelone (UTI), treatment by physician’s choice (TPC), and vinorelbine (VIN).

**Results:**

The network meta-analysis included seven trials. Results showed that second- or later-line patients treated with ERI had statistically longer overall survival versus TPC (hazard ratio [HR]: 0.81; credible interval [CrI]: 0.66–0.99) or GEM+VIN (0.62; 0.42–0.90) and statistically longer progression-free survival versus TPC (0.76; 0.64–0.90), but statistically shorter progression-free survival versus CAP+IXA (1.40; 1.17–1.67) and CAP+UTI (1.61; 1.23–2.12). In triple negative breast cancer, ERI had statistically longer overall survival versus CAP (0.70; 0.54–0.90); no statistical differences in progression-free survival were observed in triple negative breast cancer.

**Conclusions:**

This network meta-analysis suggests that ERI may provide an overall survival benefit in the overall locally advanced breast cancer/metastatic breast cancer populations and triple negative breast cancer subgroup compared to standard treatments. These findings support the use of ERI in second- or later-line treatment of patients with locally advanced breast cancer/metastatic breast cancer.

**Supplementary Information:**

The online version contains supplementary material available at 10.1186/s12885-021-08446-8.

## Background

The prognosis of locally advanced breast cancer (LABC)/metastatic breast cancer (MBC) depends heavily on patient and disease characteristics (e.g., tumor size, extent of lymph node involvement, sites of metastases, hormone receptor and human epidermal growth factor receptor 2 (HER2) status, and presence of an inflammatory component) [1, 2]. Treatment of LABC/MBC in the second- or later-line (2 L+) setting aims to provide maximum control of symptoms, prevent serious complications, prolong survival, delay disease progression, and maintain quality of life [3]. Selection of treatment in these lines depends on several factors, including hormone receptor (HR) and HER2 status, aggressiveness of the disease, type of prior therapy, and response to prior therapy [3].

Eribulin mesylate (ERI; Halaven®) is a microtubule inhibitor indicated for the treatment of LABC/MBC in patients who have previously received at least two chemotherapy (ChT) regimens for MBC in the United States (US), and for the treatment of LABC/MBC in patients who have progressed after at least one ChT for advanced disease in the European Union (EU). Approval for 2 L+ treatment was granted by the European Medicines Agency (EMA) in 2011 [4], and approval as a third- or later-line (3 L+) treatment for MBC was granted by the US Food and Drug Administration (FDA) in 2010 [5]. In both regions, patients should have received prior treatment with anthracyclines or taxanes, unless they were not suitable. ERI has a novel mode of action that is distinct from other tubulin-targeting agents such as taxanes and vinca alkaloids; it exclusively binds to the growing positive ends of tubulin without affecting its normal function, which might explain how ERI is able to overcome taxane resistance and have a potentially wider clinical effect [6].

A systematic literature review (SLR) was conducted to identify and synthesize available randomized controlled trial (RCT) evidence on the efficacy and safety of ChTs used in patients who have received one or more previous systemic therapies in the LABC/MBC setting. Bayesian network meta-analysis (NMA) was then used to compare the relative efficacy and safety of ERI as a 2 L+ treatment for LABC/MBC versus other ChTs in the overall population and in subgroups of triple negative breast cancer (TNBC) and HR-positive/HER2-negative populations.

## Methods

### Identification and selection of studies

The review followed methodology outlined in a pre-specified study protocol and adhered to Preferred Reporting Items for Systematic Reviews and Meta-Analyses (PRISMA) guidelines [7, 8]. Systematic searches were conducted to identify peer-reviewed RCTs published from 1 January 2007 to 22 March 2019 in Embase, MEDLINE (via PubMed), and the Cochrane Library. Search strategies included a combination of controlled vocabulary including relevant medical subject headings and free-text terms such as ‘breast cancer’, ‘breast neoplasm’, ‘metastasis’, and ‘locally advanced’. The full search strategies describing the population, study design, and treatment search parameters are presented in Additional file 1. Conference proceedings of five key meetings from the 2 years prior to preparation of this manuscript (2018 to 2019) were searched, including the American Society of Clinical Oncology (ASCO), the European Society of Medical Oncology (ESMO), the Japan Society of Clinical Oncology, the Japan Society of Medical Oncology, and the International Society for Pharmacoeconomics and Outcomes Research (ISPOR). Conference proceedings from the above meetings were hand-searched using free-text terms for ‘breast cancer’. Additionally, bibliographies of identified SLRs were also searched manually to confirm that all relevant studies were included.

Screening was conducted using pre-defined eligibility criteria that were based on population, interventions, comparisons, outcomes, and study design (PICOS; Table 1). At the title/abstract level, all abstracts were screened by one investigator, and a second reviewer examined 10% of exclusions to validate screening quality of the first reviewer. Full-text screening was conducted by two independent investigators, with discrepancies resolved by a third investigator. The primary outcomes of interest were overall survival (OS), progression-free survival (PFS), rate of treatment discontinuation due to adverse events (TDAEs), and rate of serious adverse events (SAEs). Comparators of interest for inclusion in the NMA were monotherapy and combination strategies of: capecitabine (CAP), gemcitabine (GEM), ixabepilone (IXA), utidelone (UTI), vinorelbine (VIN), and treatment by physician’s choice (TPC).

**Table 1:** PICOS Criteria for Study Selection

PICOS	Inclusion Criteria	Exclusion Criteria
Population	Patients with LABC or MBC who had received at least one prior therapy •. LABC or MBC defined as stage IV, any T, and N, M1^a^ •. Target populations were HER2-negative or TNBC, but HER2-positive populations were also included	•. Early disease (stage I–III) •. First-line treatment
Interventions	•. Eribulin mesylate (Halaven®) •. Carboplatin (Paraplatin®) •. Cisplatin (Platinol®; Platinol®-AQ) •. Cyclophosphamide (Cytoxan®; Neosar®) •. Doxorubicin (Adriamycin®; Rubex®) •. Doxorubicin liposomal (Doxil®) •. Epirubicin (Ellence®) •. Capecitabine (Xeloda®) •. Fluorouracil (Adrucil®) •. Gemcitabine (Gemzar®) •. Methotrexate (amethopterin) •. Docetaxel (Taxotere®) •. Ixabepilone (Ixempra®) •. Paclitaxel (Taxol®; Onxal™) •. Protein-bound paclitaxel (Abraxane®) •. Vinorelbine (Navelbine®)	Interventions not of interest or administered in combination with interventions of interest
Comparators	BSC, placebo, or all therapies listed above as monotherapy or in combination with other treatments for LABC/MBC	
Outcomes	•. Efficacy: OS, PFS, response (including ORR, CR, PR, SD, PD) •. Safety: AEs, SAEs, discontinuation, and death	Studies not reporting any outcomes of interest
Study Design	RCTs	Observational studies (including but not limited to case controls, prospective and retrospective cohorts, cross-sectional studies, single-arm trials, SLRs, NMAs, dose-escalation studies)
Countries	Any	NA

*Abbreviations*: *AE* Adverse event, *BSC* Best supportive care, *CR* Complete response, *HER2* Human epidermal growth factor receptor 2, *LABC* Locally advanced breast cancer, *MBC* Metastatic breast cancer, *NA* Not applicable, *NMA* Network meta-analysis, *ORR* Objective response rate, *OS* Overall survival, *PD* Progressive disease, *PFS* Progression-free survival, *PICOS* Population, interventions, comparisons, outcomes, and study design, *PR* Partial response, *RCT* Randomized controlled trial, *SAE* Serious adverse event, *SD* Stable disease, *SLR* Systematic literature review, *TNBC* Triple-negative breast cancer

^a^ Any size, with or without nearby lymph node involvement, spread to distant organs or distal lymph nodes

### Data extraction and assessment

Data from RCTs that met the eligibility criteria were extracted by one reviewer, and a second reviewer validated the extraction for accuracy. Data fields of interest included study design (e.g., trial phase and blinding), methods, sample size, disease and patient characteristics, intervention details (e.g., dosage, schedule), study follow-up and assessment time periods, and efficacy and safety outcomes. The quality of RCTs was assessed using the Centre for Reviews and Dissemination tool according to the National Institute for Health and Care Excellence (NICE) Guide to the Methods of Technology Appraisal [9].

Evidence identified from the SLR was assessed to determine the feasibility of comparing efficacy and safety estimates via an NMA. The network of evidence for each outcome of interest was assessed, and the need for and validity of any assumptions required to connect the network was evaluated. Specifically, the feasibility assessment evaluated whether RCTs were comparable with regards to factors that impact the relative effect of each of the treatments on each outcome, including patient-level characteristics, relevancy of interventions (comparable dosage and schedules), availability of outcome data overall and by subgroup (e.g., TNBC, HR-positive/HER2-negative, number of prior lines of therapy), outcome definitions, and timepoints at which outcomes were assessed.

**Fig. 1:**
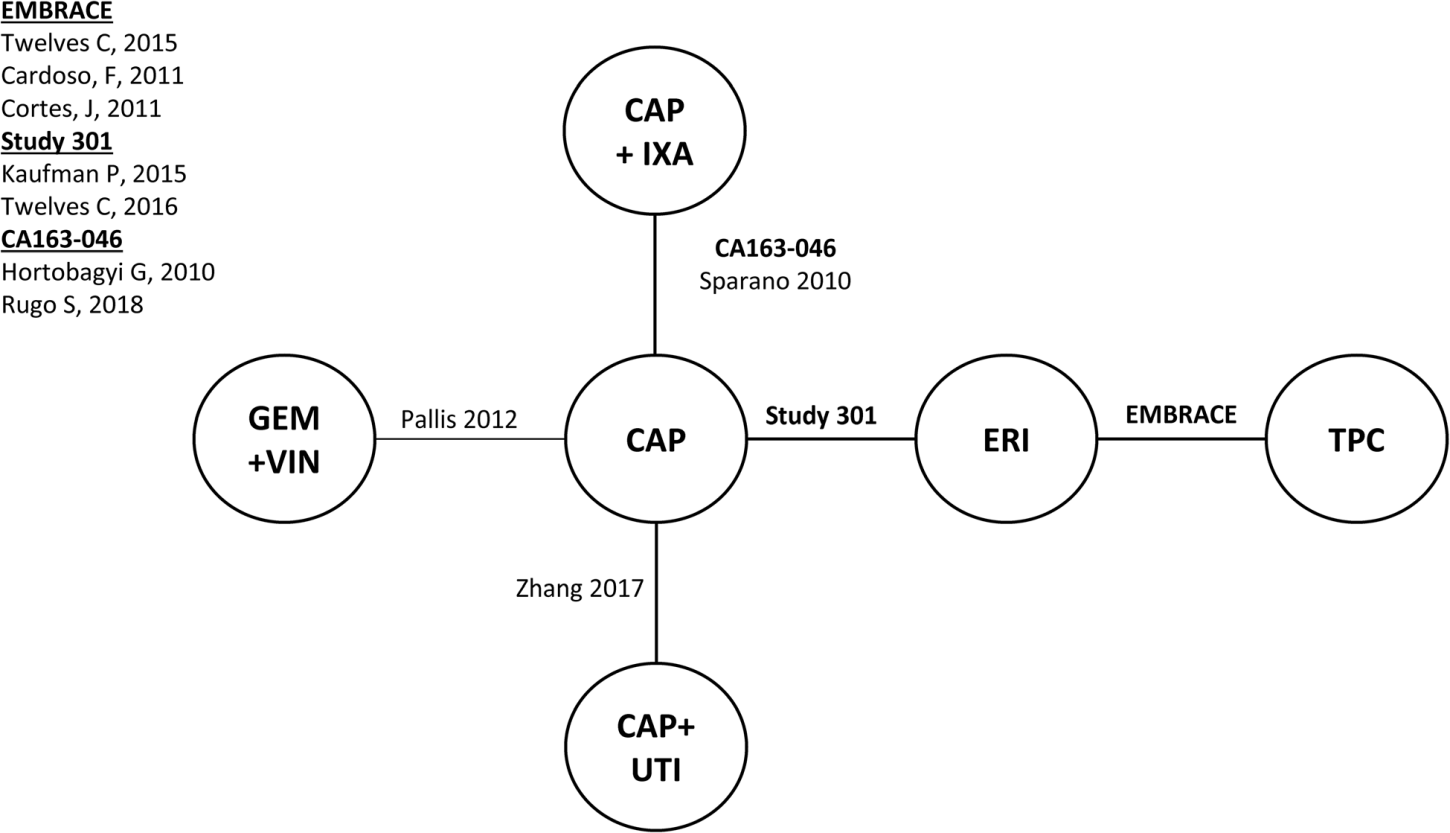
Network Diagram: Overall Survival (Base-case Analysis). Abbreviations: CAP = capecitabine; ERI = eribulin; GEM = gemcitabine; IXA = ixabepilone; TPC = treatment by physician’s choice; UTI = utidelone; VIN = vinorelbine

**Fig. 2:**
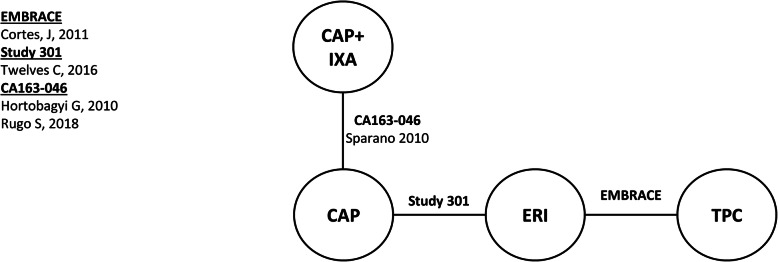
Network Diagram: Overall Survival (TNBC Subgroup Analysis). Abbreviations: CAP = capecitabine; ERI = eribulin; IXA = ixabepilone; TNBC = triple negative breast cancer; TPC = treatment by physician’s choice

**Fig. 3:**
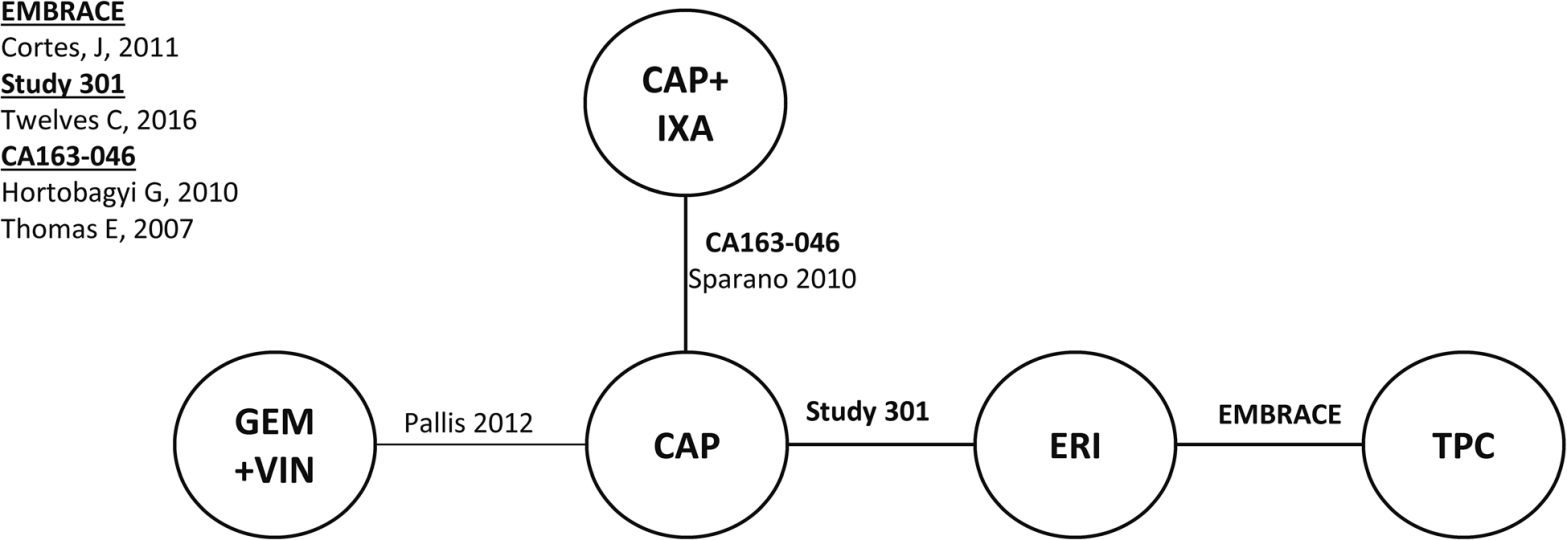
Network Diagram: Overall Survival (HER2-negative Subgroup Analysis). Abbreviations: CAP = capecitabine; ERI = eribulin; GEM = gemcitabine; IXA = ixabepilone; TPC = treatment by physician’s choice; VIN = vinorelbine

**Fig. 4:**
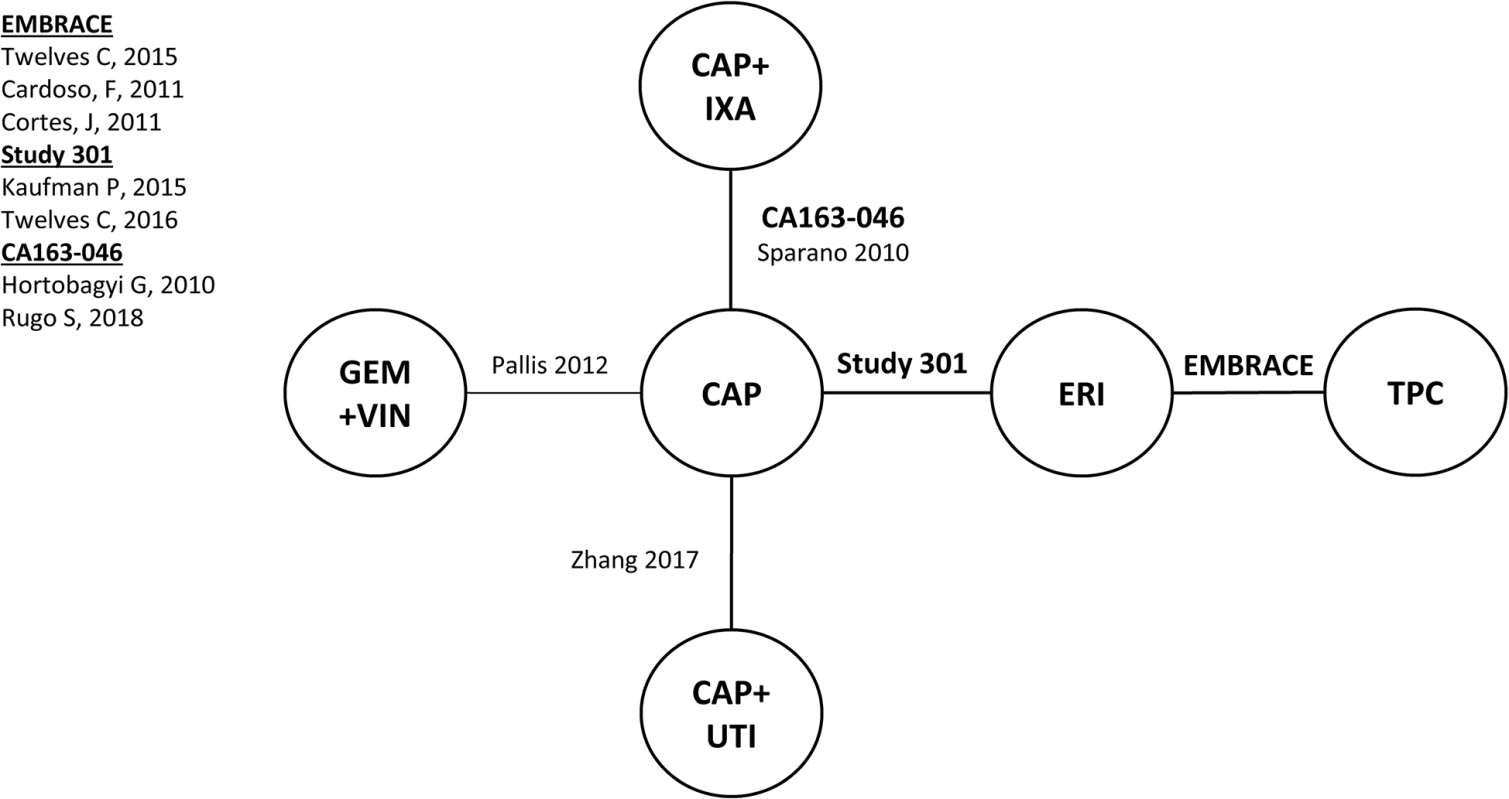
Network Diagram: Progression-free Survival (Base-case Analysis). Abbreviations: CAP = capecitabine; ERI = eribulin; GEM = gemcitabine; IXA = ixabepilone; TPC = treatment by physician’s choice; UTI = utidelone; VIN = vinorelbine

**Fig. 5:**
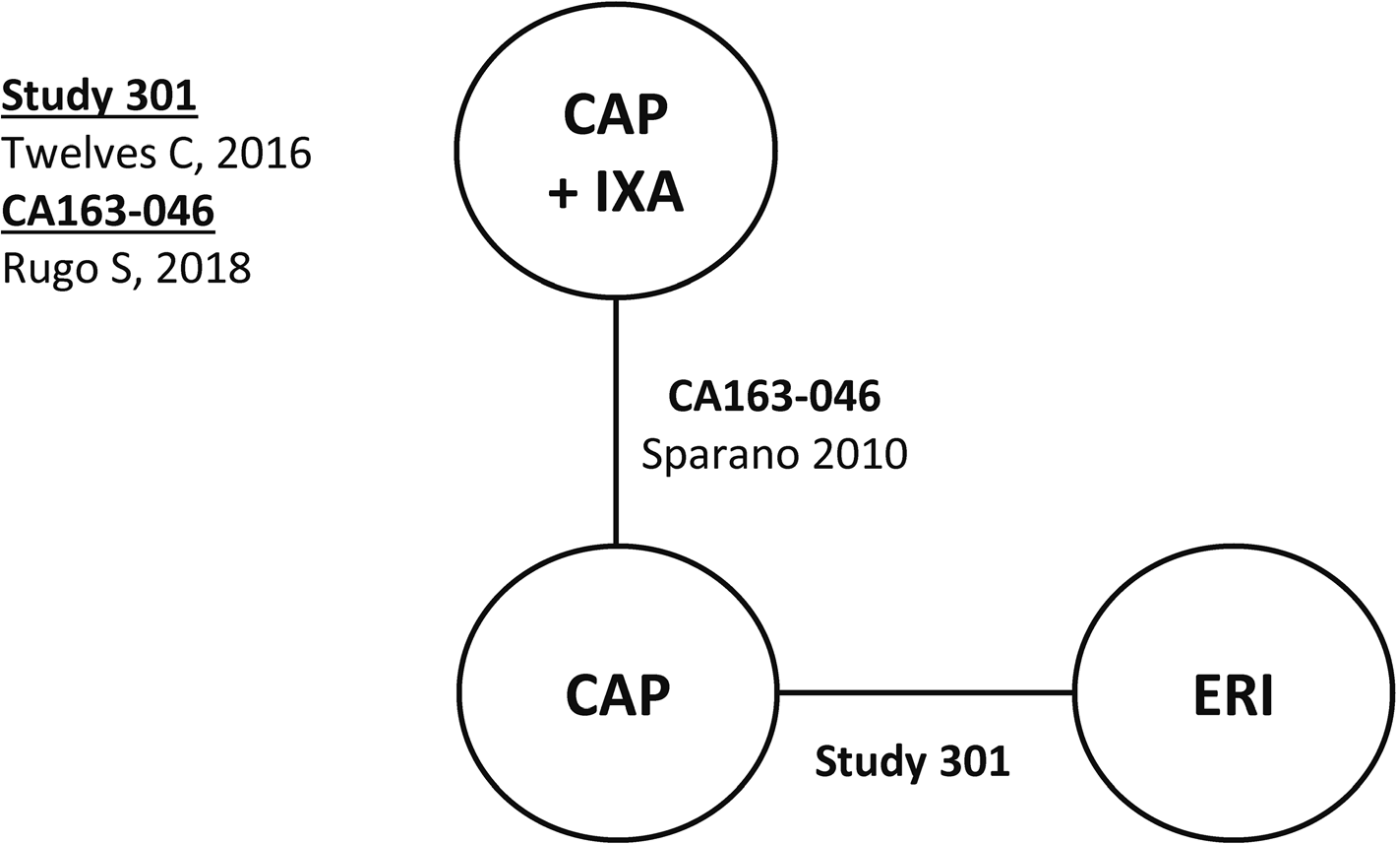
Network Diagram: Progression-free Survival (TNBC Subgroup Analysis). Abbreviations: CAP = capecitabine; ERI = eribulin; IXA = ixabepilone; TNBC = triple negative breast cancer; TPC = treatment by physician’s choice

**Fig. 6:**
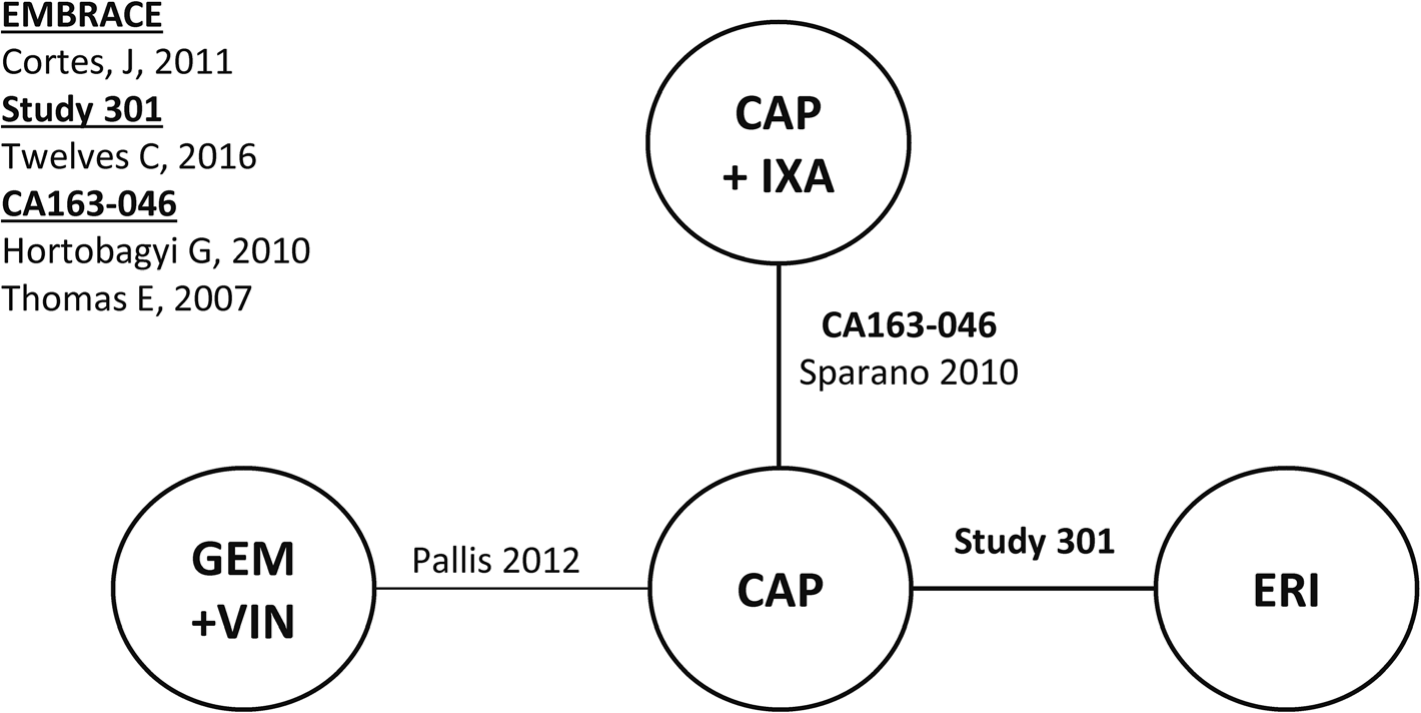
Network Diagram: Progression-free Survival (HER2-negative Subgroup Analysis). Abbreviations: CAP = capecitabine; ERI = eribulin; GEM = gemcitabine; IXA = ixabepilone; TPC = treatment by physician’s choice; VIN = vinorelbine

**Fig. 7:**
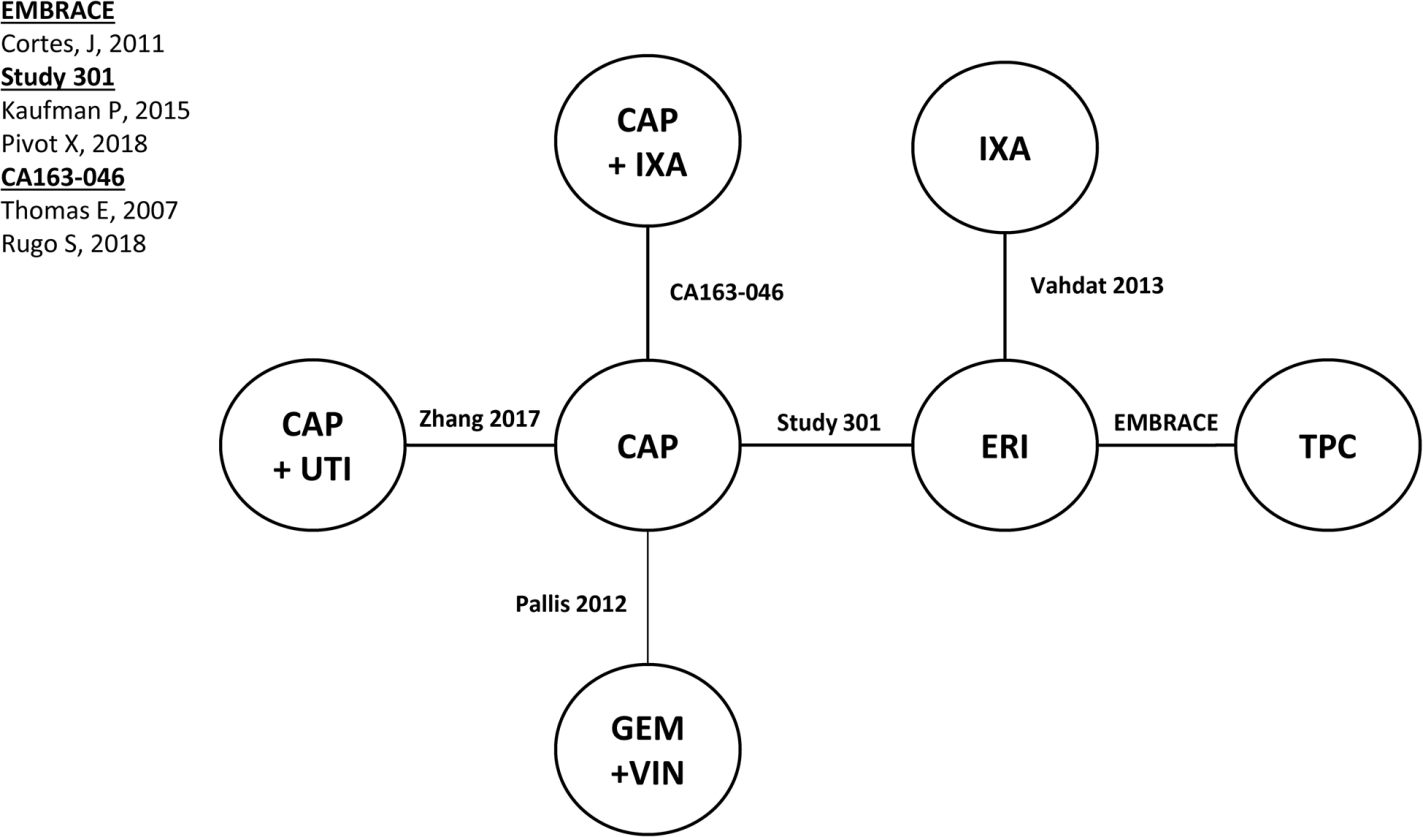
Network Diagram: Discontinuation due to AEs (Base-case Analysis). Abbreviations: AE = adverse event; CAP = capecitabine; ERI = eribulin; GEM = gemcitabine; IXA = ixabepilone; TPC = treatment by physician’s choice; UTI = utidelone; VIN = vinorelbine

**Fig. 8:**
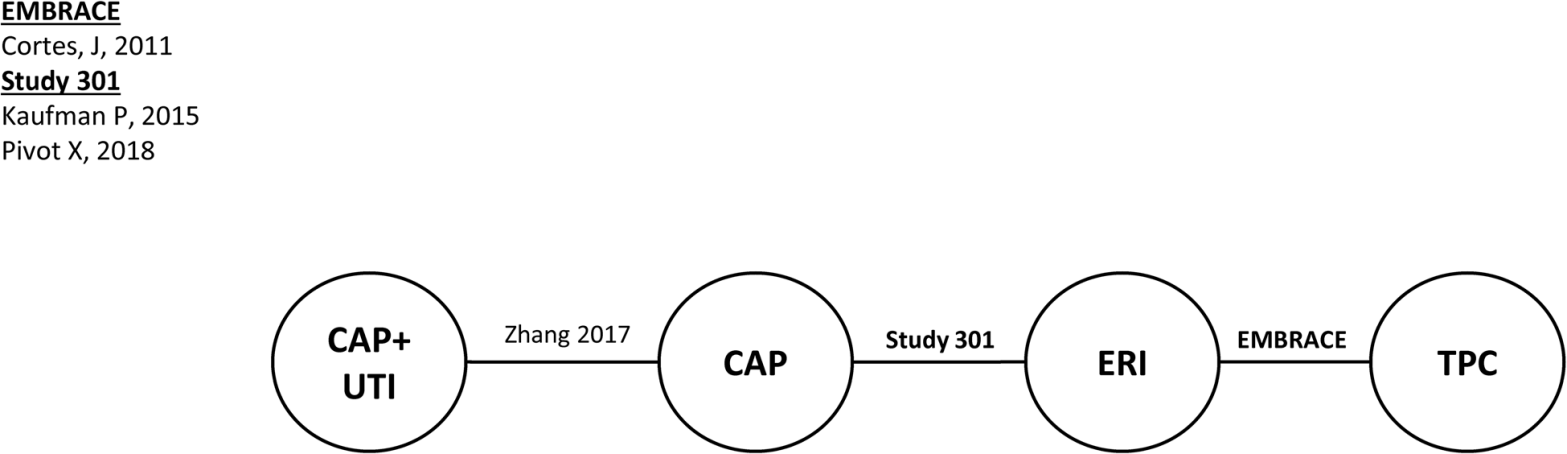
Network Diagram: SAEs (Base-case Analysis). Abbreviations: CAP = capecitabine; ERI = eribulin; SAE = serious adverse event; TPC = treatment by physician’s choice; UTI = utidelone

Connected networks that included ERI and at least a subset of comparators for key efficacy and safety outcomes were identified and are presented in Figs. 1, 2, 3, 4, 5, 6, 7, 8. Direct head-to-head efficacy and safety comparisons versus ERI were available for CAP and TPC, and other comparators were compared indirectly. Studies reporting treatment with VIN monotherapy, docetaxel (DOC) monotherapy, GEM+DOC, and CAP+DOC were identified by the SLR, but did not connect to the networks.

### Descriptive analyses and Bayesian NMA approach

Following recommendations from the feasibility assessment, the Bayesian NMA included scenario analyses to explore the potential impact of suspected effect modifiers from clinical heterogeneity across trial populations and within subgroups. For example, we conducted subgroup analyses for TNBC and HR-positive/HER2-negative patients. Subgroup analyses by line of therapy were also planned for patients receiving treatment in the 2 L+ versus 3 L+ setting, but ultimately not conducted due to a lack of data allowing for this stratification, as discussed further below (see “Results”).

In each analysis, estimates for ERI versus each comparator of interest were obtained using long-established Bayesian NMA techniques [10, 11]. A fixed-effect model was used as the primary analysis approach for each outcome due to limited heterogeneity between trials; however, if two or more studies were available for at least one treatment comparison that showed some evidence of heterogeneity (i.e., when pairwise I^2^ ≥ 25%, where I^2^ is the percentage of variation in the treatment estimates between studies beyond change), a random-effect model was employed.

All Bayesian analyses were conducted in OpenBUGS 3.2.3, with 100,000 iterations for “burn-in” and 100,000 iterations for the posterior inference. Each NMA provided an estimate of the relative treatment effect (e.g., a hazard ratio [HR] for OS and PFS or an odds ratio [OR] for SAEs, along with a 95% credible interval [CrI]). Throughout the results, the terminology ‘statistical difference’ or ‘statistically’ are used in instances where CrIs for HRs or ORs do not include 1.

## Results

### Systematic literature review

#### Study selection

**Fig. 9:**
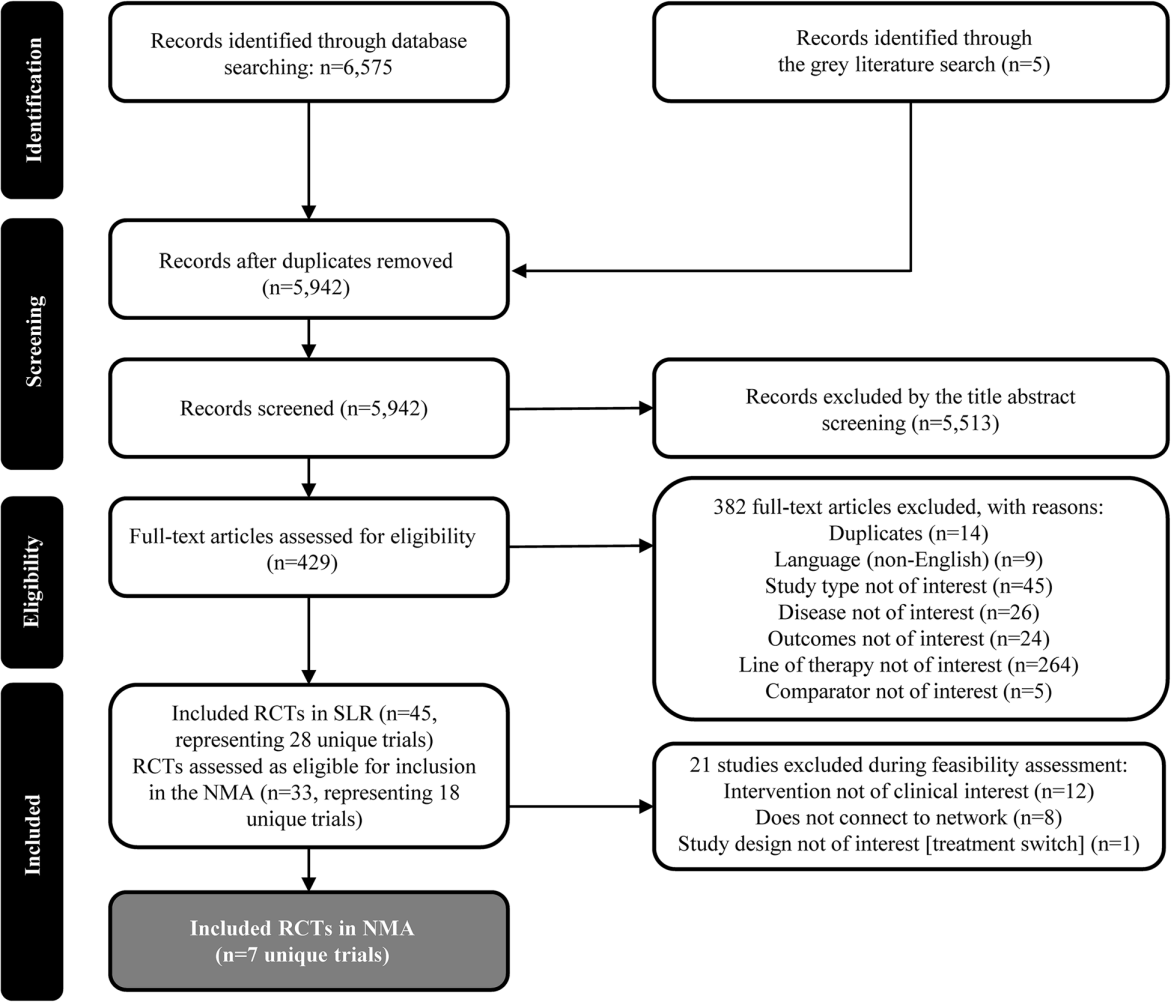
PRISMA Flow Diagram of the SLR and NMA. Abbreviations: NMA = network meta-analysis; RCT = randomized controlled trial; SLR = systematic literature review

The SLR searches identified 5942 records for title/abstract screening after duplicate removal. Of these, 429 articles were screened for full-text eligibility and 45 publications representing 28 unique RCTs were included in the SLR. Of the 28 RCTs identified by the SLR, 21 trials were excluded from the NMA for the following reasons: 12 trials investigated treatments not of clinical interest for the NMA (olaparib, talazoparib, bevacizumab, cetuximab, imatinib mesylate, onartuzumab, trastuzumab, sorafenib), eight trials did not connect to the networks for any outcomes of interest, and one trial used a study design not of interest (treatment switching). Subsequently, a total of seven RCTs were included in the NMA, as presented in the PRISMA diagram in Fig. 9.

#### Treatment characteristics

An overview of the study characteristics and treatments of the seven included trials is provided in Table 2. The frequency of treatments evaluated as monotherapy or combination therapy in the seven RCTs included in the NMA were CAP (five studies), ERI (three studies), IXA (three studies), GEM (one study), UTI (one study), VIN (one study), and TPC (one study). Mechanisms of action of treatments evaluated included both antimicrotubule agents (ERI, IXA, UTI, VIN), antimetabolites (CAP, GEM), or therapeutic combinations of the two (e.g., GEM+VIN, CAP+UTI). Use of TPC involved administration of any single-agent chemotherapy, hormonal, or biological treatment approved for the treatment of cancer administered according to local practice, radiotherapy, or as symptomatic treatment alone. Four of the studies investigated combination strategies and three studies investigated use of monotherapies. Treatments evaluated in more than one RCT were each similar with respect to dosages, regimens, routes of administration, and cycle lengths.

**Table 2:** Overview of Study Characteristics of Trials Included in the NMA

Trial	Brief Patient Description	RCT Design	Treatments	N Randomized	Objectives
Study 301 *NCT00337103* Kaufman 2015 [12] Twelves 2016 [13] Cortes 2015 [14] Pivot 2018 [15]	Women with MBC who had received prior anthracycline- and taxane-based therapy	Phase III Open-label	1) ERI 2) CAP	1102	To compare ERI with CAP in patients with LABC or MBC.
EMBRACE *NCT00388726* Cortes 2011 [16] Twelves 2015 [17] Cardoso 2011 [18]	Women with heavily pre-treated (third line to fifth line) locally recurrent or MBC	Phase III Open-label	1) ERI 2) TPC: 25% VIN, 19% GEM, 18% CAP, 15% taxanes, 10% anthracyclines, 10% other chemo, 4% hormonal therapy	1102	To compare OS of women with heavily pre-treated MBC receiving ERI or real-life treatment choices.
Pallis, 2012 [19] *NCT00431106*	Women with MBC, pre-treated and/or resistant to anthracyclines and taxanes	Phase III Blinding NR	1) CAP 2) VIN + GEM	172	To demonstrate superiority of combination treatment in terms of PFS.
Vahdat, 2013 [20] *NCT00879086*	Women with locally recurrent or MBC who had received prior taxane therapy, at least one prior cytotoxic chemotherapy for advanced disease, and progressed during last anti-cancer treatment	Phase II Open-label	1) ERI 2) IXA	104	To assess the incidence of neuropathy.
CA163–046 *NCT00080301* Thomas, 2007 [21] Hortobagyi, 2010 [22] Rugo 2018 [23]	Women with LABC or MBC, pre-treated with or resistant to anthracyclines and taxanes	Phase III Open-label	1) IXA + CAP 2) CAP	752	To describe the results of OS from the CA163–046 phase III study.
CA163–048 *NCT00082433* Sparano 2010 [24] Rugo 2018 [23]	Women previously treated with an anthracycline- and taxane-containing regimen	Phase III Open-label	1) IXA + CAP 2) CAP	1221	To assess whether the combination improved survival compared with CAP monotherapy.
Zhang 2017 [25] *NCT02253459*	Female patients with MBC refractory to anthracycline and taxane	Phase III Open-label	1) UTI + CAP 2) CAP	405	To compare the efficacy and safety of UTI + CAP vs. CAP alone in patients with MBC.

*Abbreviations*: *CAP* CApecitabinek, *ERI* Eribulin, *GEM* Gemcitibine, *HER2* Human epidermal growth factor receptor 2, *IXA* Ixabepilone, *LABC* Locally advanced breast cancer, *MBC* Metastatic breast cancer, *NR* Not reported, *OS* Overall survival, *PFS* Progression-free survival, *RCT* Randomized controlled trial, *TPC* Treatment by physician’s choice, *UTI* Utidelone, *VIN* Vinorelbine

#### Patient characteristics

A total of 4494 patients were included in the seven trials. Sample sizes ranged from 104 [20] to 1221 patients [24]. All patients had LABC or MBC and had received prior treatment with anthracyclines and taxanes. Two trials enrolled metastatic patients only [19, 25] and the remainder enrolled a mix of metastatic and locally advanced patients. Trial populations were generally similar with respect to known effect modifiers, including age, sex, type of prior therapy, and performance status (Eastern Cooperative Oncology Group [ECOG] and Karnofsky Performance Status [KPS]). Average age ranged from 50 [25] to 60 years [19]. Five trials provided baseline ECOG performance scores, and most patients had ECOG scores of 0 to 1; the percentage of patients with ECOG scores of 2 was low across the trials (2% [12, 25] to 8% [16]). The remaining two trials measured baseline performance status according to KPS, and 65% [24] and 70% [22] of patients had scores of 90 to 100%. Patient data for time from diagnosis to start of treatment was not available from five of the seven RCTs.

Across the seven included trials, heterogeneity was noted in the following potential effect modifiers: HER2 status, TNBC status, and number of prior treatments. A total of 16.1% of patients had HER2-positive disease (11% [20] to 26% [25] across trials) and 21.5% had TNBC (14% [20] to 26% [12] across trials). To explore the impact of this heterogeneity, subgroup analyses for TNBC and HER2-negative patients were conducted for the four RCTs that reported outcomes by TNBC status and the five RCTs that reported outcomes by HER2-negative status. All RCTs enrolled mixed patient populations with ≥1 prior ChT; 28% of trial populations (0% [16] to 55% [19] per study) had received one prior treatment. The distribution of prior treatment across studies was: ≥1 prior line (three trials [19, 20, 22]), 1–2 prior lines (one trial [24]), 1–3 prior lines (one trial [12]), 1–4 prior lines (one trial [25]), and 2–5 prior lines (one trial [16]). In the trial that enrolled mixed lines of therapy inclusive of first-line patients, only one patient had not received any prior chemotherapy (< 1%) [12]. While variability was observed for number of prior lines of therapy, NMA subgroup analysis of this characteristic was not feasible due to limited outcome stratification by line of therapy (e.g., two prior lines versus three or more prior lines as planned).

#### Outcome assessments

Of the seven trials, six reported OS, six reported PFS, six reported discontinuation due to AEs, and three reported SAEs. All trials provided definitions for the primary outcome assessments (OS and PFS), and no differences were observed across the trials. For safety outcomes, all studies assessed adverse events (AEs) using the National Cancer Institute Common Terminology Criteria for Adverse Events (CTCAE version 3 or 4), except for one study which did not report a definition.

#### Risk-of-Bias assessments

Most RCTs were assessed as having a low risk of bias (Additional file 2); however, some concerns were noted for one trial, in which differences in baseline performance status were noted in addition to its open-label design [22].

### Network meta-analysis

Eight sets of analyses are presented, including two base-case analyses for OS and PFS, two TNBC subgroup analyses for OS and PFS, two HR-positive/HER2-negative subgroup analyses for OS and PFS, and two safety analyses.

#### Overall survival

**Fig. 10:**
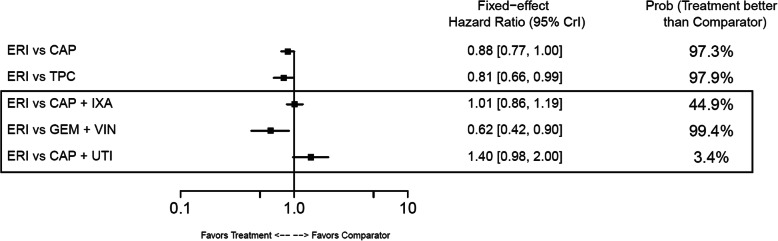
Forest Plot of Overall Survival Treatment Comparison. Estimates derived only from indirect comparisons shown in black box. Included references: Study 301 (Kaufman 2015; Twelves 2016), EMBRACE (Twelves 2015; Cardoso 2011; Cortes 2011), CA163–046 (Hortobagyi 2010; Rugo 2018), Sparano 2010, Pallis 2012, Zhang 2017. Abbreviations: CAP = capecitabine; CrI = credible interval; ERI = eribulin; GEM = gemcitabine; IXA = ixabepilone; TPC = treatment by physician’s choice; UTI = utidelone; VIN = vinorelbine

**Fig. 11:**
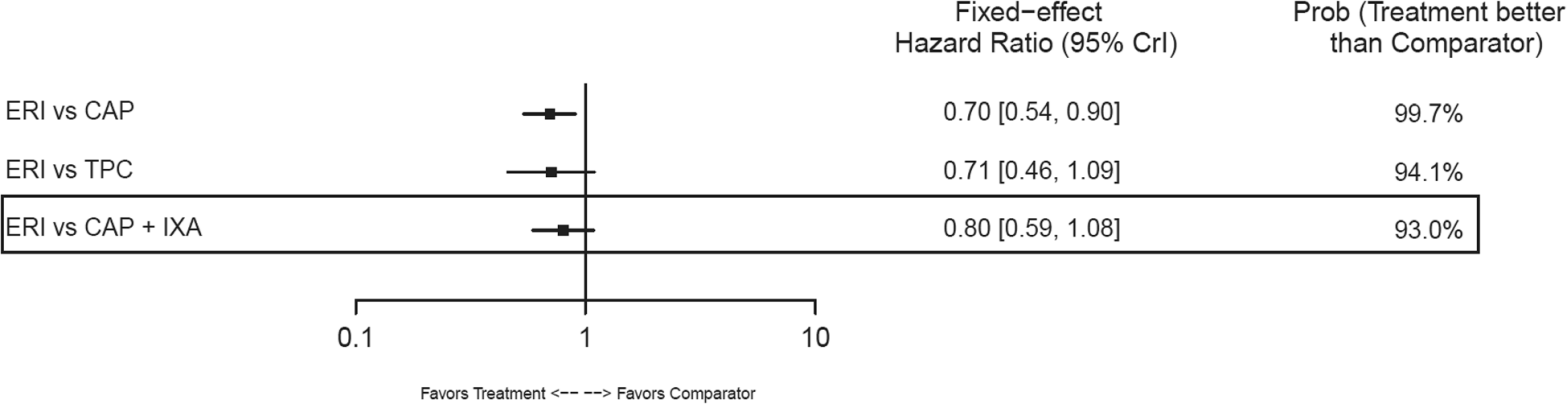
Forest Plot of Overall Survival Treatment Comparison: TNBC Subgroup Analysis. Estimates derived only from indirect comparisons shown in black box. Included references: Study 301 (Twelves 2016), EMBRACE (Cortes 2011), CA163–046 (Hortobagyi 2010; Rugo 2018), Sparano 2010. Abbreviations: CAP = capecitabine; CrI = credible interval; ERI = eribulin; IXA = ixabepilone; TNBC = triple negative breast cancer; TPC = treatment by physician’s choice.

**Fig. 12:**
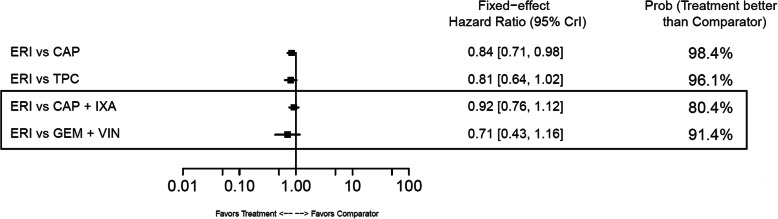
Forest Plot of Overall Survival Treatment Comparison: HER2– Subgroup Analysis. Estimates derived only from indirect comparisons shown in black box. Included references: Study 301 (Twelves 2016), EMBRACE (Cortes 2011), CA163–046 (Hortobagyi 2010; Rugo 2018), Sparano 2010, Pallis 2012. Abbreviations: CAP = capecitabine; CrI = credible interval; ERI = eribulin; GEM = gemcitabine; HER2 = human epidermal growth factor receptor 2; IXA = ixabepilone; TPC = treatment by physician’s choice; VIN = vinorelbine

In the base-case analysis, ERI-treated patients had statistically longer OS compared with those treated with TPC (HR: 0.81; 95% CrI: 0.66–0.99) or GEM+VIN (HR: 0.62; 95% CrI: 0.42–0.90) (Fig. 10). No other base-case comparisons were statistically different. In the TNBC subgroup, ERI had statistically longer OS compared with CAP (HR: 0.70; 95% CrI: 0.54–0.90) (Fig. 11). Point estimates for OS across the remaining TNBC subgroup comparisons favored ERI; however, none of these comparisons were statistically different. In the HR-positive/HER2-negative subgroup, ERI-treated patients also had statistically longer OS than those treated with CAP (HR: 0.84; 95% CrI: 0.71–0.98). Point estimates for OS in HR-positive/HER2-negative subgroups favored ERI for all remaining comparisons; however, similar to the TNBC subgroup results, these comparisons were not statistically different (Fig. 12).

#### Progression-free survival

**Fig. 13:**
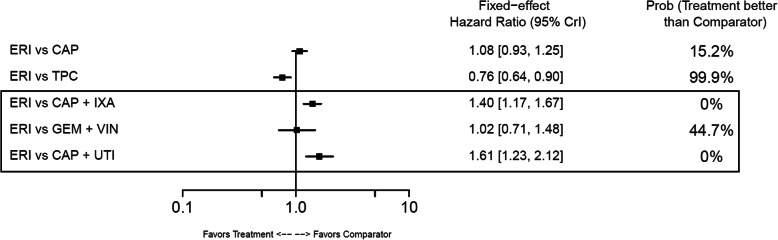
Forest Plot of Progression-free Survival Treatment Comparison. Estimates derived only from indirect comparisons shown in black box. Included references: Study 301 (Kaufman 2015; Twelves 2016), EMBRACE (Twelves 2015; Cardoso 2011; Cortes 2011), CA163–046 (Hortobagyi 2010; Rugo 2018), Sparano 2010, Pallis 2012, Zhang 2017. Abbreviations: CAP = capecitabine; CrI = credible interval; ERI = eribulin; GEM = gemcitabine; IXA = ixabepilone; TPC = treatment by physician’s choice; UTI = utidelone; VIN = vinorelbine

**Fig. 14:**
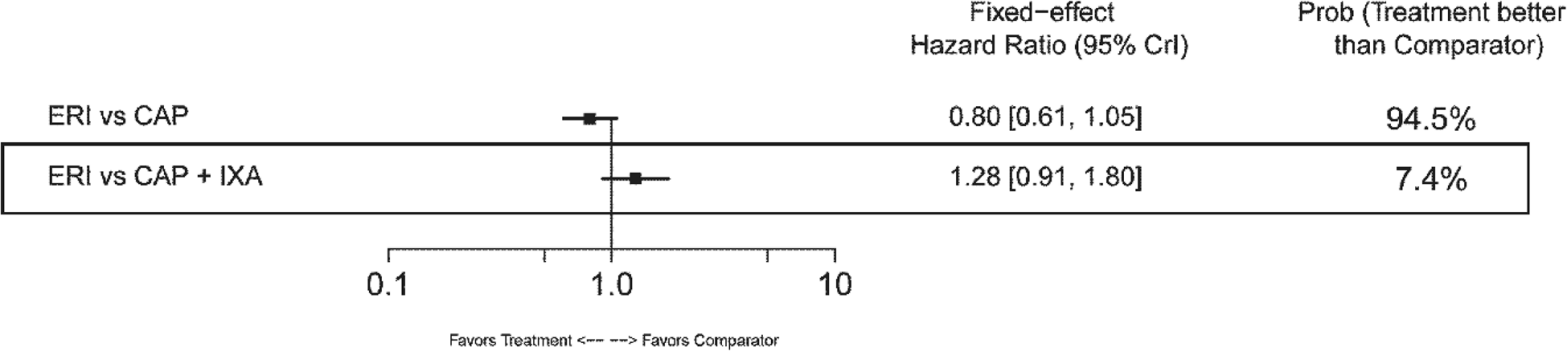
Forest Plot of Progression-free Survival Treatment Comparison: TNBC Subgroup Analysis. Estimates derived only from indirect comparisons shown in black box. Included references: Study 301 (Kaufman 2015), CA163–046 (Rugo 2018), Sparano 2010. Abbreviations: CAP = capecitabine; CrI = credible interval; ERI = eribulin; IXA = ixabepilone; TNBC = triple negative breast cancer

**Fig. 15:**
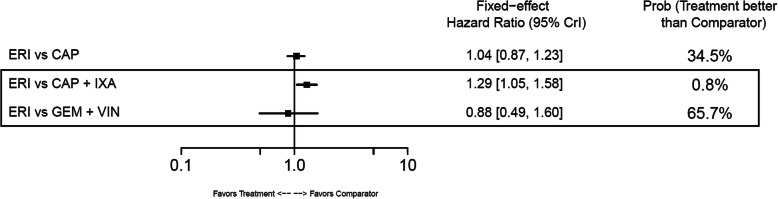
Forest Plot of Progression-free Survival Treatment Comparison: HER2– Subgroup Analysis. Estimates derived only from indirect comparisons shown in black box. Included references: Study 301 (Twelves 2016), CA163–046 (Hortobagyi 2010; Thomas 2007), Sparano 2010, Pallis 2012. Abbreviations: CAP = capecitabine; CrI = credible interval; ERI = eribulin; GEM = gemcitabine; HER2 = human epidermal growth factor receptor 2; IXA = ixabepilone; VIN = vinorelbine

In the base-case analysis, ERI was associated with a significantly longer PFS compared with TPC (HR: 0.76; 95% CrI: 0.64–0.90) and a significantly shorter PFS versus CAP+IXA (HR: 1.40; 95% CrI: 1.17–1.67) and CAP+UTI (HR:1.61; 95% CrI: 1.23–2.12) (Fig. 13). No statistical differences for ERI versus comparators were observed in the TNBC subgroup (Fig. 14), whose network had only two comparisons. In the HR-positive/HER2-negative subgroup, which comprised three treatment comparisons, patients treated with CAP+IXA had statistically longer PFS than those treated with ERI (HR: 1.29; 95% CrI:1.05–1.58) (Fig. 15).

#### Safety

**Fig. 16:**
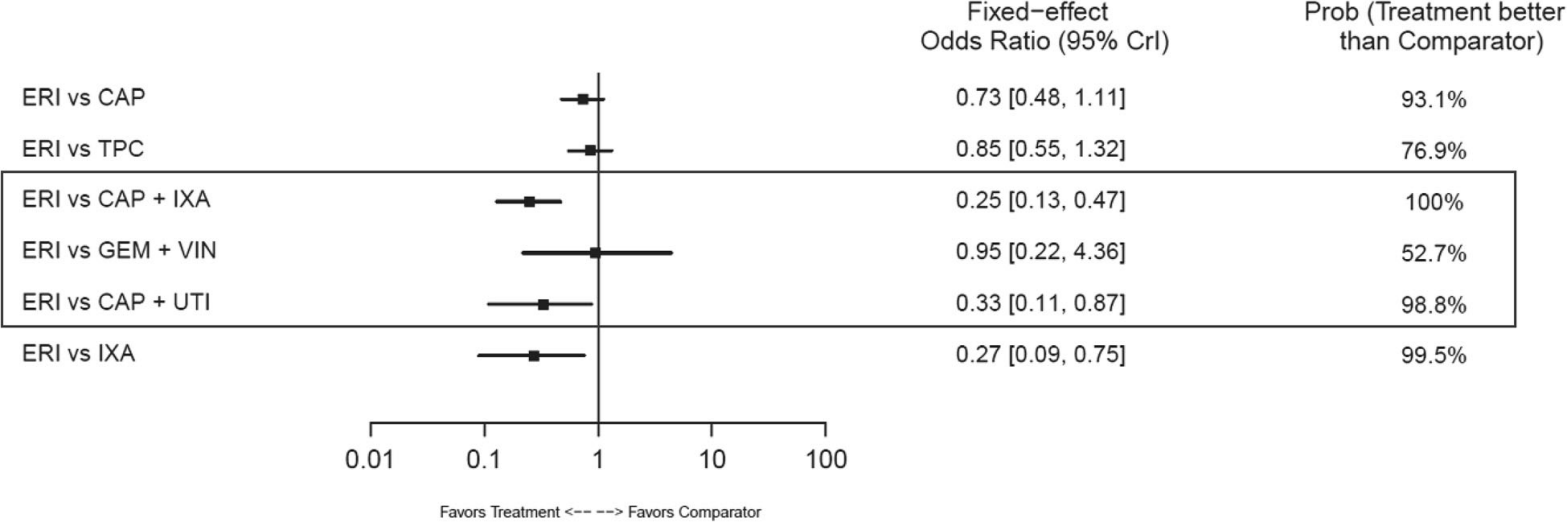
Forest Plot of Discontinuation Due to AEs: Safety Analysis. Estimates derived only from indirect comparisons shown in black box. Included references: Study 301 (Kaufman 2015, Pivot 2018), EMBRACE (Cortes 2011), CA163–046 (Thomas 2007; Rugo 2018), Pallis 2012, Vahdat 2013, Zhang 2017. Abbreviations: AE = adverse event; CAP = capecitabine; CrI = credible interval; ERI = eribulin; GEM = gemcitabine; IXA = ixabepilone; TPC = treatment by physician’s choice; UTI = utidelone; VIN = vinorelbine

**Fig. 17:**
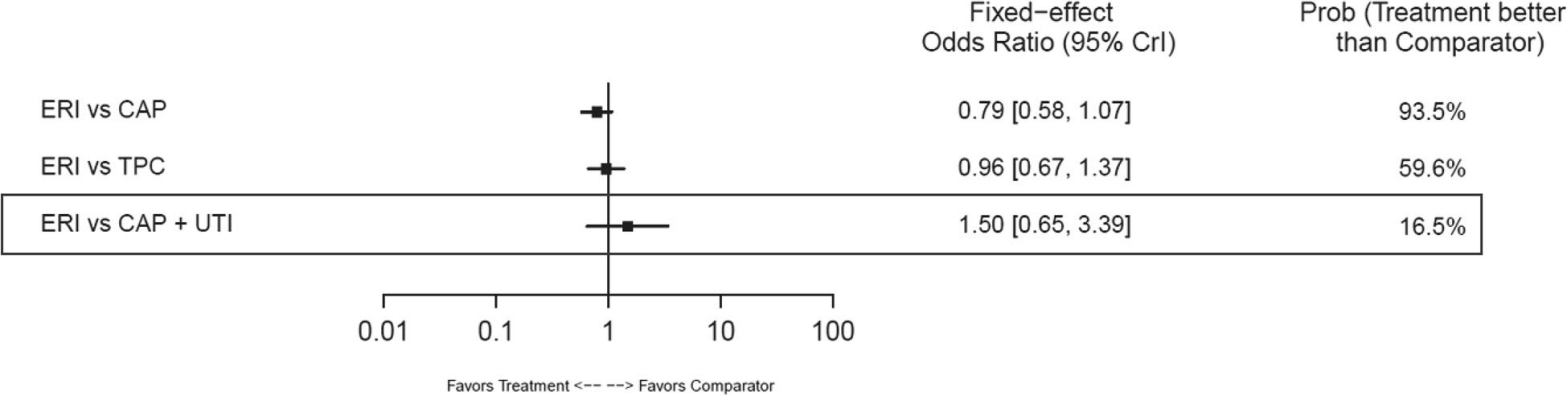
Forest Plot of SAEs: Safety Analysis. Estimates derived only from indirect comparisons shown in black box. Included references: Study 301 (Kaufman 2015, Pivot 2018), EMBRACE (Cortes 2011), Zhang 2017. Abbreviations: CAP = capecitabine; CrI = credible interval; ERI = eribulin; SAE = serious adverse event; TPC = treatment by physician’s choice; UTI = utidelone

In safety outcome analyses, there was a trend toward ERI reducing treatment discontinuation due to AEs across all comparators, with statistical advantages compared with CAP+IXA (HR: 0.25; 95% CrI: 0.13–0.47), CAP+UTI (HR: 0.33; 95% CrI: 0.11–0.87), and IXA (HR: 0.27; 95% CrI: 0.09–0.75). No statistical differences between the other comparisons were observed (Fig. 16). No statistical differences were found between ERI and any comparator for SAEs (Fig. 17).

## Discussion

This SLR and NMA estimated the relative efficacy and safety of ERI as a 2 L+ treatment for LABC/MBC versus other ChTs in the overall population and in subgroups of TNBC and HR-positive/HER2-negative patients. The SLR and NMA were conducted in accordance with published guidelines and based on well-established Bayesian methodology [11, 26]. Three of the seven RCTs included in the NMA directly compared ERI to other active treatments [12–14, 16–18]. Using Bayesian NMA, it was possible to pool and indirectly compare RCT evidence on treatments for which no head-to-head trials were available. For example, outcomes from four trials providing data for CAP + IXA (two trials), CAP + UTI, and GEM + VIN were indirectly compared to ERI for OS and PFS base-case analyses.

While baseline patient characteristics across the seven RCTs in the NMA were generally similar for most patient characteristics such as age and performance status, variability was noted for known effect modifiers of TNBC status, HR-positive/HER2-negative status, and number of lines of therapy. Analyses of these subgroups was warranted to explore differences in treatment effect in these specific populations versus the broader overall population; however, not all trials provided outcome data stratified by TNBC status or by HR-positive/HER2-negative status. Differences across the included trials were also noted for number of prior lines of therapy. Given heterogeneity of treatment effect originating from number of prior lines of therapy may exist, the NMA sought to examine the differences, but limited OS and PFS data stratified by line of therapy across the included trials prevented this analysis. The inability to conduct these analyses is a limitation of the NMA, as the number of prior lines of therapy is a potential effect modifier in LABC/MBC and stratification by line of therapy has been integral to the methodology of existing NMAs in the disease area [27, 28].

In the base-case network, the NMA showed statistically significant prolongation of OS for ERI-treated patients compared to TPC- or GEM+VIN-treated patients. The NMA also showed a trend for longer OS in CAP+UTI-treated patients over ERI-treated patients, but this difference was not statistically significant. In TNBC and HR-positive/HER2-negative subgroups, the NMA showed statistically significant OS benefit for treatment with ERI versus CAP. Similar to the OS results, significantly longer PFS was observed in ERI-treated patients compared with TPC; however, in the base-case PFS network, the NMA found that treatment with CAP+UTI or CAP+IXA had significantly prolonged PFS compared to ERI. Further, in the HR-positive/HER2-negative subpopulation, CAP+IXA also had longer PFS compared with ERI.

Between the execution of the searches on 22 March 2019 and the drafting of this manuscript, an additional relevant publication, Yuan et al. 2019 [29], has been identified that would contribute to the NMA networks. Since this trial was published outside of the existing search dates, it was not eligible for inclusion in the current analysis; however, a systematic update to the SLR was conducted on 8 January 2021 that confirmed the publication’s eligibility for inclusion, and an updated feasibility assessment and NMA are in progress. Yuan et al. 2019 investigates a head-to-head comparison of two key comparators—ERI and VIN. The inclusion of a connection to VIN monotherapy in the analysis would expand the existing base-case network by opening additional indirect comparisons to other eligible comparators including: docetaxel [30], GEM + docetaxel [31], CAP + docetaxel [32], paclitaxel [33], and nab-paclitaxel [34, 35].

The most important limitation of this NMA was the lack of data to control for population-level differences in determinants of response to treatment. For example, the duration of response to prior lines of therapy and quality of prior response are known to be important effect modifiers in LABC/MBC, along with line of therapy, as noted earlier. However, these analyses were ultimately not possible due to lack of stratification of the published RCT data by these factors. This NMA was also constrained by the small number of treatment comparisons available for outcomes of interest both for the base-case and for subgroups. A larger data pool may have allowed for indirect comparisons of ERI with other ChTs of interest and may have helped reduced CrIs for those comparisons which were possible, but it should be noted that additional RCTs would also potentially introduce additional heterogeneity into the networks. The strengths of this review were first its methodology, which was systematic, reproducible, and adherent to PRISMA guidelines and second, its thorough comparison and assessment of the evidence base for inclusion, with relevant scenario analyses conducted when permitted by available data.

## Conclusion

This NMA of available RCTs suggests that ERI may provide a favorable OS benefit in overall LABC/MBC populations and TNBC subgroups compared to standard treatments. Specifically, the NMA suggests that ERI provides a statistically significant OS benefit compared with TPC and GEM+VIN in 2 L+ treatment of patients with LABC/MBC and compared with CAP in TNBC and HR-positive/HER2-negative subgroups. ERI shows significantly lower rates of discontinuation due to AEs than CAP+IXA, CAP+UTI, and IXA. These NMA findings further support the clinical value of treatment with ERI in LABC/MBC.

## Supplementary Information

**Additional file 1.** Systematic Literature Review Search Strategies. Tables containing the terms and yields for searches conducted in Embase, MEDLINE, and Cochrane library from 1 January 2007 to 22 March 2019.

**Additional file 2.** Risk of Bias Assessment Results. Figure depicting the risk of bias assessment results of individual included studies assessed using the Centre for Reviews and Dissemination tool.

## Data Availability

Not applicable.

## Abbreviations

2 L+: Second- or later-line
3 L+: Third- or later-line.
AE: Adverse event
ASCO: American Society of Clinical Oncology
CAP: Capecitabine
ChT: Chemotherapy
Crl: Credible interval
CTCAE: Common Terminology Criteria for Adverse Events
DOC: Docetaxel
ECOG: Eastern Cooperative Oncology Group
EMA: European Medicines Agency
ERI: Eribulin mesylate
ESMA: European Society of Medical Oncology
EU: European Union
FDA: Food and Drug Administration
GEM: Gemcitabine
HER2: Human epidermal growth factor receptor 2
HR: Hazard ratio
HR: Hormone receptor
ISPOR: International Society for Pharmacoeconomics and Outcomes Research
IXA: Ixabepilone
KPS: Karnofsky Performance Status
LABC: Locally advanced breast cancer
MBC: Metastatic breast cancer
NICE: National Institute for Health and Care Excellence
NMA: Network meta-analysis
OR: Odds ratio
OS: Overall survival
PFS: Progression-free survival
PICOS: Population, interventions, comparisons, outcomes, and study design
PRISMA: Preferred Reporting Items for Systematic Reviews and Meta-Analyses
RCT: Randomized controlled trial
SAE: Serious adverse event
SLR: Systematic literature review
TDAE: Treatment discontinuation due to adverse events
TNBC: Triple negative breast cancer
TPC: Treatment by physician’s choice
US: United States
UTI: Utidelone
VIN: Vinorelbine
