# Supplementary material for: Network meta-analysis of eribulin versus other chemotherapies used as second- or later-line treatment in locally advanced or metastatic breast cancer
Source: BMC Cancer. 2021 Jun 30;21:758. doi: 10.1186/s12885-021-08446-8 (PMC8244131; doi:10.1186/s12885-021-08446-8)
Supplement: Supplementary file 1 — Additional file 1. Systematic Literature Review Search Strategies. Tables containing the terms and yields for searches conducted in Embase, MEDLINE, and Cochrane library from 1 January 2007 to 22 March 2019. [file 12885_2021_8446_MOESM1_ESM.docx]

Supplementary Table S1. Embase: SLR Update (2018–2019)

| **Topic** | **Search Criteria** | **Search Algorithm** | **Yield**  **(March 22, 2019)** |
| --- | --- | --- | --- |
| Population | 1 | ‘breast cancer’/exp OR ‘breast neoplasm’/exp OR ‘breast carcinoma’/exp OR ‘mammary cancer’/exp OR ‘breast tumour’/exp OR ‘breast tumor’/exp | 558,778 |
|  | 2 | ‘metastasis’/exp OR ‘metastatic’:ti,ab OR ‘metastasize’:ti,ab OR ‘metastasis’:ti,ab OR ‘metastases’:ti,ab OR ‘advanced’:ti,ab OR ‘stage 4’:ti,ab OR ‘stage IV’:ti,ab OR ‘terminal’:ti,ab | 1,702,905 |
|  | 3 | ‘locally advanced’:ti,ab AND (‘recurrent’:ti,ab OR ‘inoperable’:ti,ab OR ‘incurable’:ti,ab OR ‘unresectable’:ti,ab) | 8,074 |
|  | 4 | #1 AND (#2 OR #3) | 159,764 |
| Interventions | 5 | ‘eribulin’:ti,ab OR ‘halaven’:ti,ab OR ‘carboplatin’:ti,ab OR ‘paraplatin’:ti,ab OR ‘cisplatin’:ti,ab OR ‘platinol’:ti,ab OR ‘platinol-AQ’:ti,ab OR ’platinol AQ’:ti,ab OR ‘cyclophoshamide’:ti,ab OR ‘cytoxan’:ti,ab OR ‘neosar’:ti,ab OR ‘doxorubicin’:ti,ab OR ‘adriamycin’:ti,ab OR ‘rubex’:ti,ab OR ‘liposomal doxorubicin’:ti,ab OR ‘doxil’:ti,ab OR ‘epirubicin’:ti,ab OR ‘ellence’:ti,ab OR ‘capecitabine’:ti,ab OR ‘xeloda’:ti,ab OR ‘fluorouracil’:ti,ab OR ‘5?fu’:ti,ab OR ‘5 fluorouracil’:ti,ab OR ‘5-fluorouracil’:ti,ab OR ‘adrucil’:ti,ab OR ‘gemcitabine’:ti,ab OR ‘gemzar’:ti,ab OR ‘methotrexate’:ti,ab OR ‘amethopterin’:ti,ab OR ‘docetaxel’:ti,ab OR ‘taxotere’:ti,ab OR ‘ixabepilone’:ti,ab OR ‘ixempra’:ti,ab OR ‘paclitaxel’:ti,ab OR ‘taxol’:ti,ab OR ‘onxal’:ti,ab OR ‘albumin-bound paclitaxel’:ti,ab OR ‘albumin bound paclitaxel’:ti,ab OR ‘nab paclitaxel’:ti,ab OR ‘abraxane’:ti,ab OR ‘vinorelbine’:ti,ab OR ‘navelbine’:ti,ab | 313,184 |
| Study design | 6 | ‘clinical trial’/exp OR ‘randomized controlled trial’/exp OR ‘controlled clinical trial’/exp OR ‘multicenter study’/exp OR ‘phase 3 clinical trial’/exp OR ‘phase 4 clinical trial’/exp OR ‘randomization’/exp OR 'single blind procedure'/exp OR ‘double blind procedure’/exp OR ‘crossover procedure’/exp OR ‘placebo’/exp OR ‘randomi*ed controlled trial*’:ti,ab OR rct:ti,ab OR (‘random* allocat*’ OR ‘allocated randomly’):ti,ab OR (allocated NEXT/2 random):ti,ab OR ‘single blind*’:ti,ab OR ‘double blind*’:ti,ab OR ((treble OR triple) NEXT/1 blind*):ti,ab OR placebo*:ti,ab OR ‘prospective study’/exp | 2,140,007 |
|  | 7 | ‘case study’/exp OR ‘case report’:ti,ab OR (‘abstract report’ OR letter):ti,ab OR ‘conference proceeding’:pt OR ‘editorial’:pt OR ‘letter’:pt OR ‘note’:pt | 539,627 |
|  | 8 | #6 NOT #7 | 2,112,733 |
| Population, interventions, Study designs | 9 | #4 AND #5 AND #8 | 6,929 |
| Limits | 10 | 'animal'/exp NOT 'human'/exp | 5,204,680 |
|  | 11 | #9 NOT #10 | 6,889 |
|  | 12 | #11 AND [english]/lim | 6,609 |
|  | 13 | #12 AND [2018-2019]/py | 344 |
|  | 14 | #13 AND [conference abstract]/lim | 192 |
| Articles | 15 | #13 NOT #14 | 152 |
| Conference abstracts (2014-2018) | 16 | #14 AND [conference abstract]/lim AND 'journal of clinical oncology'/jt AND [2018-2019]/py | 65 |
|  | 17 | #14 AND [conference abstract]/lim AND 'annals of oncology'/jt AND [2018-2019]/py | 0 |
|  | 18 | #14 AND [conference abstract]/lim AND 'value in health'/jt AND [2018-2019]/py | 2 |
|  | 19 | #14 AND [conference abstract]/lim AND 'international journal of clinical oncology'/jt AND [2018-2019]/py | 0 |
|  |  | Conference abstracts (from databases) | 67 |
|  |  | Articles | 152 |
|  |  | TOTAL | 219 |

**Supplementary Table S2. Embase: New Targeted Therapies (2007–2019)**

| **Topic** | **Search Criteria** | **Search Algorithm** | **Yield**  **(March 22, 2019)** |
| --- | --- | --- | --- |
| Population | 1 | ‘breast cancer’/exp OR ‘breast neoplasm’/exp OR ‘breast carcinoma’/exp OR ‘mammary cancer’/exp OR ‘breast tumour’/exp OR ‘breast tumor’/exp | 558,778 |
|  | 2 | ‘metastasis’/exp OR ‘metastatic’:ti,ab OR ‘metastasize’:ti,ab OR ‘metastasis’:ti,ab OR ‘metastases’:ti,ab OR ‘advanced’:ti,ab OR ‘stage 4’:ti,ab OR ‘stage IV’:ti,ab OR ‘terminal’:ti,ab | 1,702,905 |
|  | 3 | ‘locally advanced’:ti,ab AND (‘recurrent’:ti,ab OR ‘inoperable’:ti,ab OR ‘incurable’:ti,ab OR ‘unresectable’:ti,ab) | 8,074 |
|  | 4 | #1 AND (#2 OR #3) | 159,976 |
| Intervention | 5 | ‘talazoparib’/exp OR ‘talzenna’:ti,ab OR ‘olaparib’/exp OR ‘lynparza’:ti,ab | 3,830 |
| Study design | 6 | ‘clinical trial’/exp OR ‘randomized controlled trial’/exp OR ‘controlled clinical trial’/exp OR ‘multicenter study’/exp OR ‘phase 3 clinical trial’/exp OR ‘phase 4 clinical trial’/exp OR ‘randomization’/exp OR 'single blind procedure'/exp OR ‘double blind procedure’/exp OR ‘crossover procedure’/exp OR ‘placebo’/exp OR ‘randomi*ed controlled trial*’:ti,ab OR rct:ti,ab OR (‘random* allocat*’ OR ‘allocated randomly’):ti,ab OR (allocated NEXT/2 random):ti,ab OR ‘single blind*’:ti,ab OR ‘double blind*’:ti,ab OR ((treble OR triple) NEXT/1 blind*):ti,ab OR placebo*:ti,ab OR ‘prospective study’/exp | 2,143,160 |
|  | 7 | ‘case study’/exp OR ‘case report’:ti,ab OR (‘abstract report’ OR letter):ti,ab OR ‘conference proceeding’:pt OR ‘editorial’:pt OR ‘letter’:pt OR ‘note’:pt | 540,176 |
|  | 8 | #6 NOT #7 | 2,125,864 |
| Population, interventions, Study designs | 9 | #4 AND #5 AND #8 | 250 |
| Limits | 10 | 'animal'/exp NOT 'human'/exp | 5,205,725 |
|  | 11 | #9 NOT #10 | 249 |
|  | 12 | #11 AND [english]/lim | 246 |
|  | 13 | #12 AND [2007-2019]/py | 246 |
|  | 14 | #13 AND [conference abstract]/lim | 86 |
| Articles | 15 | #13 NOT #14 | 160 |
| Conference abstracts (2014-2018) | 16 | #14 AND [conference abstract]/lim AND 'journal of clinical oncology'/jt AND [2018-2019]/py | 7 |
|  | 17 | #14 AND [conference abstract]/lim AND 'annals of oncology'/jt AND [2018-2019]/py | 0 |
|  | 18 | #14 AND [conference abstract]/lim AND 'value in health'/jt AND [2018-2019]/py | 1 |
|  | 19 | #14 AND [conference abstract]/lim AND 'international journal of clinical oncology'/jt AND [2018-2019]/py | 0 |
|  |  | Conference abstracts (from databases) | 8 |
|  |  | Articles | 160 |
|  |  | TOTAL | 168 |

**Supplementary Table S3. MEDLINE (via pubmed.com) SLR Update (2018–2019)**

| **Topic** | **Search Criteria** | **Search Algorithm** | **Yield**  **(22 Mar 2019)** |
| --- | --- | --- | --- |
| Population | 1 | “breast neoplasms”[MH] | 272,769 |
|  | 2 | “metastatic”[TIAB] OR “metastasize”[TIAB] OR “metastasis”[TIAB] OR “metastases”[TIAB] OR “advanced”[TIAB] OR “stage 4”[TIAB] OR “stage IV”[TIAB] OR “terminal”[TIAB] | 1,171,269 |
|  | 3 | “locally advanced”[TIAB] AND (“recurrent” [TIAB] OR “inoperable”[TIAB] OR “incurable”[TIAB] OR “unresectable”[TIAB]) | 4,524 |
|  | 4 | #1 AND (#2 OR #3) | 61,192 |
| Interventions | 5 | Eribulin[TIAB] OR halaven[TIAB] OR Carboplatin[TIAB] OR paraplatin[TIAB] OR cisplatin[TIAB] OR platinol[TIAB] OR platinol-AQ[TIAB] OR platinol AQ[TIAB] OR Cyclophosphamide[TIAB] OR Cytoxan[TIAB] OR neosar[TIAB] OR Doxorubicin[TIAB] OR adriamycin[TIAB] OR rubex[TIAB] OR liposomal doxorubicin[TIAB] OR doxil[TIAB] OR Epirubicin[TIAB] OR ellence[TIAB] OR Capecitabine[TIAB] OR xeloda[TIAB] OR Fluorouracil[TIAB] OR 5?fu[TIAB] OR 5 fluorouracil[TIAB] OR 5-fluorouracil[TIAB] OR adrucil[TIAB] OR Gemcitabine[TIAB] OR gemzar[TIAB] OR Methotrexate[TIAB] OR amethopterin[TIAB] OR Docetaxel[TIAB] OR taxotere[TIAB] OR Ixabepilone[TIAB] OR ixempra[TIAB] OR Paclitaxel[TIAB] OR taxol[TIAB] OR onxal[TIAB] OR albumin-bound paclitaxel[TIAB] OR albumin bound paclitaxel[TIAB] OR nab paclitaxel[TIAB] OR abraxane[TIAB] OR Vinorelbine[TIAB] OR navelbine[TIAB] | 247,439 |
| Study design | 6 | Randomized controlled trials as topic[MH] OR Randomized controlled trial[PT] OR Random allocation[MH] OR Double blind method[MH] OR Single blind method[MH] OR Clinical trial[MH] OR Clinical Trial, Phase I[PT] OR Clinical Trial, Phase II[PT] OR Clinical Trial, Phase III[PT] OR Clinical Trial, Phase IV[PT] OR Controlled Clinical Trial[PT] OR Randomized Controlled Trial[PT] OR Multicenter Study[PT] OR Clinical trial[PT] OR Clinical Trials as Topic[MH] OR Clinical trial*[TW] OR Single blind*[TW] OR double blind*[TW] OR treble blind*[TW] OR triple blind*[TW] OR Placebos[MH] OR Placebo*[TW] OR Randomly allocated[TW] OR allocated random*[TW] | 1,522,660 |
|  | 7 | Case report[TW] OR Letters as topic[MH] OR Historical article[TW] | 641,463 |
|  | 8 | #6 NOT #7 | 1,516,553 |
| Population, intervention, study design | 9 | #4 AND #5 AND #8 | 3,958 |
| Limits | 10 | Animal[MH] | 22,136,355 |
|  | 11 | Human[MH] | 17,582,382 |
|  | 12 | #10 NOT #11 | 4,553,973 |
|  | 13 | #9 NOT #12 | 3,957 |
|  | 14 | #13 AND English[Language] | 3,731 |
|  | 15 | #14 AND (“2018”[Date – Publication] : “2019”[Date – Publication]) | 45 |

**Supplementary Table S4. MEDLINE (via pubmed.com): New Targeted Therapies (2007–2019)**

| **Topic** | **Search Criteria** | **Search Algorithm** | **Yield**  **(22 Mar 2019)** |
| --- | --- | --- | --- |
| Population | 1 | “breast neoplasms”[MH] | 273,079 |
|  | 2 | “metastatic”[TIAB] OR “metastasize”[TIAB] OR “metastasis”[TIAB] OR “metastases”[TIAB] OR “advanced”[TIAB] OR “stage 4”[TIAB] OR “stage IV”[TIAB] OR “terminal”[TIAB] | 1,172,232 |
|  | 3 | “locally advanced”[TIAB] AND (“recurrent” [TIAB] OR “inoperable”[TIAB] OR “incurable”[TIAB] OR “unresectable”[TIAB]) | 4,529 |
|  | 4 | #1 AND (#2 OR #3) | 61,261 |
| Interventions | 5 | “Talazoparib”[TIAB] OR “talzenna”[TIAB] OR “olaparib”[TIAB] OR “lynparza”[TIAB] | 896 |
| Study design | 6 | Randomized controlled trials as topic[MH] OR Randomized controlled trial[PT] OR Random allocation[MH] OR Double blind method[MH] OR Single blind method[MH] OR Clinical trial[MH] OR Clinical Trial, Phase I[PT] OR Clinical Trial, Phase II[PT] OR Clinical Trial, Phase III[PT] OR Clinical Trial, Phase IV[PT] OR Controlled Clinical Trial[PT] OR Randomized Controlled Trial[PT] OR Multicenter Study[PT] OR Clinical trial[PT] OR Clinical Trials as Topic[MH] OR Clinical trial*[TW] OR Single blind*[TW] OR double blind*[TW] OR treble blind*[TW] OR triple blind*[TW] OR Placebos[MH] OR Placebo*[TW] OR Randomly allocated[TW] OR allocated random*[TW] | 1,523,638 |
|  | 7 | Case report[TW] OR Letters as topic[MH] OR Historical article[TW] | 641,799 |
|  | 8 | #6 NOT #7 | 1,517,987 |
| Population, intervention, study design | 9 | #4 AND #5 AND #8 | 29 |
| Limits | 10 | Animal[MH] | 22,144,969 |
|  | 11 | Human[MH] | 17,589,716 |
|  | 12 | #10 NOT #11 | 4,555,253 |
|  | 13 | #9 NOT #12 | 29 |
|  | 14 | #13 AND English[Language] | 28 |
|  | 15 | #14 AND (“2007”[Date – Publication] : “2019”[Date – Publication]) | 28 |

**Supplementary Table S5. Cochrane Library: SLR Update (2018–2019)**

| **Topic** | **Search Criteria** | **Search Algorithm** | **Yield**  **(March 22, 2019)** |
| --- | --- | --- | --- |
| Population | 1 | [mh “breast neoplasms”] OR “breast cancer”:ti,ab,kw OR “breast neoplasm”:ti,ab,kw OR “breast carcinoma”:ti,ab,kw OR “mammary cancer”:ti,ab,kw OR “breast tumour”:ti,ab,kw OR “breast tumour”:ti,ab,kw | 26,916 |
|  | 2 | [mh “neoplasm metastasis”] OR metastatic:ti,ab OR metastasize:ti,ab OR metastasis:ti,ab OR metastases:ti,ab OR advanced:ti,ab OR stage 4:ti,ab OR stage IV:ti,ab OR terminal:ti,ab | 79,736 |
|  | 3 | “locally advanced”:ti,ab AND (recurrent:ti,ab OR inoperable:ti,ab OR incurable:ti,ab OR unresectable:ti,ab) | 933 |
|  | 4 | #1 AND (#2 OR #3) | 9,916 |
| Interventions | 5 | “eribulin”:ti,ab OR “Halaven”:ti,ab OR “carboplatin”:ti,ab OR “paraplatin”:ti,ab OR “cisplatin”:ti,ab OR “platinol”:ti,ab OR “platinol-AQ”:ti,ab OR “platinol AQ”:ti,ab OR “cyclophosphamide”:ti,ab OR “Cytoxan”:ti,ab OR “neosar”:ti,ab OR “doxorubicin”:ti,ab OR “adriamycin”:ti,ab OR “rubex”:ti,ab OR “liposomal doxorubicin”:ti,ab OR “doxil”:ti,ab OR “epirubicin”:ti,ab OR “ellence”:ti,ab OR “capecitabine”:ti,ab OR “xeloda”:ti,ab OR fluorouracil:ti,ab OR 5 fluorouracil:ti,ab OR “adrucil”:ti,ab OR “gemcitabine”:ti,ab OR “gemzar”:ti,ab OR “methotrexate”:ti,ab OR “amethopterin”:ti,ab OR “docetaxel”:ti,ab OR “taxotere”:ti,ab OR “ixabepilone”:ti,ab OR “ixempra”:ti,ab OR “paclitaxel”:ti,ab OR “taxol”:ti,ab OR “onxal”:ti,ab OR albumin-bound paclitaxel:ti,ab OR albumin bound paclitaxel:ti,ab OR nab paclitaxel:ti,ab OR abraxane:ti,ab OR “vinorelbine”:ti,ab OR “navelbine”:ti,ab | 35,562 |
| Population and intervention | 6 | #4 AND #5 | 3,737 |
| Study design | 7 | #6 in Trials | 3,729 |
| Limits | 8 | #9 Publication Year from 2018 to 2019 | 97 |

**Supplementary Table S6. Cochrane Library: New Targeted Therapies (2007–2019)**

| **Topic** | **Search Criteria** | **Search Algorithm** | **Yield**  **(March 22, 2019)** |
| --- | --- | --- | --- |
| Population | 1 | [mh “breast neoplasms”] OR “breast cancer”:ti,ab,kw OR “breast neoplasm”:ti,ab,kw OR “breast carcinoma”:ti,ab,kw OR “mammary cancer”:ti,ab,kw OR “breast tumour”:ti,ab,kw OR “breast tumour”:ti,ab,kw | 26,916 |
|  | 2 | [mh “neoplasm metastasis”] OR metastatic:ti,ab OR metastasize:ti,ab OR metastasis:ti,ab OR metastases:ti,ab OR advanced:ti,ab OR stage 4:ti,ab OR stage IV:ti,ab OR terminal:ti,ab | 79,736 |
|  | 3 | “locally advanced”:ti,ab AND (recurrent:ti,ab OR inoperable:ti,ab OR incurable:ti,ab OR unresectable:ti,ab) | 933 |
|  | 4 | #1 AND (#2 OR #3) | 9,327 |
| Interventions | 5 | Talazoparib:ti,ab OR talzenna:ti,ab OR Olaparib:ti,ab OR Lynparza:ti,ab | 220 |
| Population and intervention | 6 | #4 AND #5 | 45 |
| Study design | 7 | #6 in Trials | 45 |
| Limits | 8 | #9 Publication Year from 2007 to 2019 | 45 |
