# Supplementary material for: Network meta-analysis of eribulin versus other chemotherapies used as second- or later-line treatment in locally advanced or metastatic breast cancer
Source: BMC Cancer. 2021 Jun 30;21:758. doi: 10.1186/s12885-021-08446-8 (PMC8244131; doi:10.1186/s12885-021-08446-8)
Supplement: Supplementary file 2 — Additional file 2. Risk of Bias Assessment Results. Figure depicting the risk of bias assessment results of individual included studies assessed using the Centre for Reviews and Dissemination tool. [file 12885_2021_8446_MOESM2_ESM.docx]

Supplementary Figure S1. Risk of Bias Assessment Results (Centre for Reviews and Dissemination Tool)


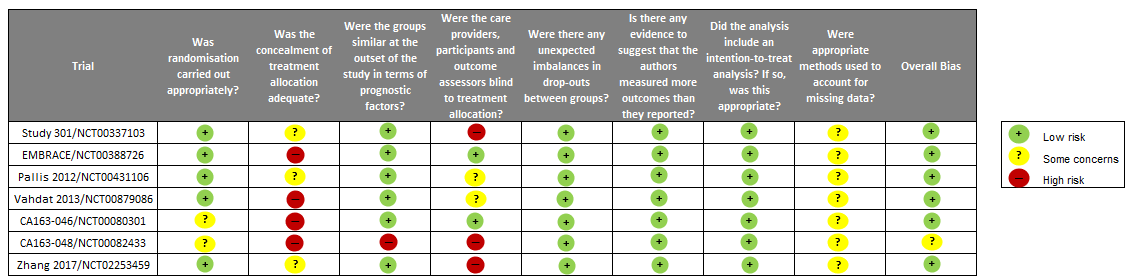


NOTE: Overall risk of bias assessment scores were graded as ‘low risk’ if seven or more of the domains were scored as ‘low’ or ‘some concerns’, as ‘some concerns’ if five or six of the domains were scored as ‘low’ or ‘some concerns’, and as ‘high risk’ if four or less of the domains were scored as ‘low’ or ‘some concerns’.
